# Exposing the Causal Effect of C-Reactive Protein on the Risk of Type 2 Diabetes Mellitus: A Mendelian Randomization Study

**DOI:** 10.3389/fgene.2018.00657

**Published:** 2018-12-20

**Authors:** Liang Cheng, He Zhuang, Shuo Yang, Huijie Jiang, Song Wang, Jun Zhang

**Affiliations:** ^1^College of Bioinformatics Science and Technology, Harbin Medical University, Harbin, China; ^2^Department of Radiology, The Second Affiliated Hospital of Harbin Medical University, Harbin, China; ^3^Department of Radiology, Longhua Hospital, Shanghai University of Traditional Chinese Medicine, Shanghai, China; ^4^Department of Rehabilitation and Pharmacy, Heilongjiang Province Land Reclamation Headquarters General Hospital, Harbin, China

**Keywords:** C-reactive protein, type 2 diabetes mellitus, causal effect, genome-wide association studies, mendelian randomization, type 1 diabetes mellitus

## Abstract

As a biomarker of inflammation, C-reactive protein (CRP) has attracted much attention due to its role in the incidence of type 2 diabetes mellitus (T2DM). Prospective studies have observed a positive correlation between the level of serum CRP and the incidence of T2DM. Recently, studies have reported that drugs for curing T2DM can also decrease the level of serum CRP. However, it is not yet clear whether high CRP levels cause T2DM. To evaluate this, we conducted a Mendelian randomization (MR) analysis using genetic variations as instrumental variables (IVs). Significantly associated single nucleotide polymorphisms (SNPs) of CRP were obtained from a genome-wide study and a replication study. Therein, 17,967 participants were utilized for the genome-wide association study (GWAS), and another 14,747 participants were utilized for the replication of identifying SNPs associated with CRP levels. The associations between SNPs and T2DM were from the DIAbetes Genetics Replication And Meta-analysis (DIAGRAM) consortium. After removing SNPs in linkage disequilibrium (LD) and T2DM-related SNPs, the four remaining CRP-related SNPs were deemed as IVs. To evaluate the pooled influence of these IVs on the risk of developing T2DM through CRP, the penalized robust inverse-variance weighted (IVW) method was carried out. The combined result (OR 1.114048; 95% CI 1.058656 to 1.172338; *P* = 0.024) showed that high levels of CRP significantly increase the risk of T2DM. In the subsequent analysis of the relationship between CRP and type 1 diabetes mellitus (T1DM), the pooled result (OR 1.017145; 95% CI 0.9066489 to 1.14225; *P* = 0.909) supported that CRP levels cannot determine the risk of developing T1DM.

## Introduction

According to the 8th edition of the International Diabetes Federation (IDF) Diabetes Atlas, the number of Diabetes Mellitus (DM) patients has continued to increase across the globe. DM includes a group of chronic metabolic diseases involving hyperglycemia (Olokoba et al., 2012; Pan et al., 2013; Shi and Hu, 2014), which, over prolonged periods, leads to injury in various tissues of the body (Atar and Hanssen, 2017; Gentile et al., 2017). The corresponding abundant fatal complications occur for patients suffering from DM for over 10 years. The IDF Diabetes Atlas reported that 425 million DM patients are adults, two-thirds (327 million) of whom are of working age. Over 90% of these patients are type 2 DM (T2DM) patients. Thus, it is urgent for us to explore a way to reduce the number of individuals with T2DM (Zou et al., 2018).

Accumulated evidence shows that intermediate phenotypes, such as body mass index (BMI) (Zhang et al., 2017; Cheng et al., 2018a), systolic blood pressure (Svensson et al., 2017), circulating uric acid (Xu et al., 2016), etc., are responsible for the onset of this type of DM (Cheng et al., 2018c). To prevent T2DM, it would be valuable to influence these intermediate phenotypes. Although there have been recent successes, a large number of potential intermediate phenotypes causing T2DM need to be identified.

Since chronic low-grade inflammation is associated with T2DM (Kohn et al., 2005), one of its markers, C-reactive protein (CRP), is frequently investigated as an intermediate phenotype that increases the risk of T2DM. Doi et al. (2005) conducted an observational study for a mean of 9.0 years on 1,759 Japanese patients without diabetes. They observed that elevated CRP concentration is a significant predictor of diabetes, independent of obesity and insulin resistance. Analogous conclusions were also observed in American and European individuals (Freeman et al., 2002; Nakanishi et al., 2003). On the other hand, after adjusting for sex, adiposity, and insulin resistance, no association between CRP and the risk of T2DM was shown in other studies (Festa et al., 2002; Krakoff et al., 2003; Thorand et al., 2007).

A recent prospective analysis and meta-analysis study (Lee et al., 2009) aimed to elucidate the role of CRP in T2DM. The high association (OR 1.49; 95% CI 1.03–2.15, *p* = 0.03) between serum CRP and the risk of diabetes was observed in a Norfolk cohort including 293 diabetes cases and 708 controls. A consistent result (RR 1.72; 95% CI 1.54–1.92) was shown in the further meta-analysis of 16 published studies involving 3,920 diabetes cases and 24,914 controls. However, no significant association (OR 1.00; 95% CI 0.66–1.51, *p* = 1.0) was obtained after adjustment for Waist-Hip Ratio (WHR), serum γ-glutamyltransferase and adiponectin. In addition, the heterogeneity of these publications (I^2^ = 52.8%) limited the conclusion in the meta-analysis.

**FIGURE 1:**
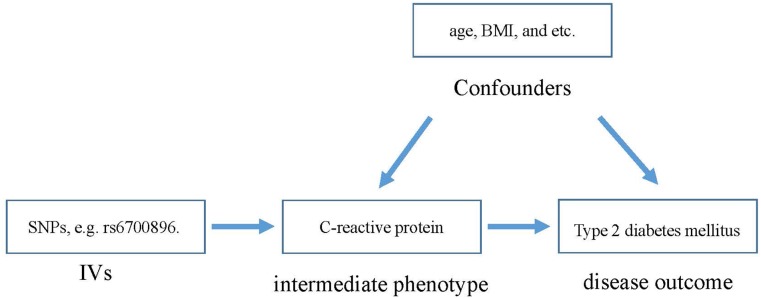
The basic principle of estimating the influence of CRP on T2DM.

The main challenge for current prospective studies is that the potential confounding factors have a great effect on the observations (Cheng et al., 2017; Zeng et al., 2018). This lack of clarity has hindered researchers in determining the association between CRP and T2DM. In addition, without evidence from randomized controlled trials (RCTs), it is hard to be sure of the role of CRP in the risk of T2DM. To this end, the Mendelian randomization (MR) method (Lawlor et al., 2008), revealing the role of a risk factor in disease etiology, is utilized here. MR is an instrumental variable (IV)-based method to infer the causality between intermediate phenotypes and disease. Genetic variants that are associated with intermediate phenotypes are introduced as IVs in MR to estimate the effect of phenotypic exposures on disease outcome (Figure 1). Due to random distribution of gene variants during gametogenesis, the confounding effect can be extremely low.

## Materials and Methods

Here, we introduced MR analysis for analyzing the causal effect of CRP on the risk of T2DM, where single nucleotide polymorphisms (SNPs) are deemed as IVs. The Mendelian randomization method is a method for testing whether a changeable exposure has a causal effect on the development of a disease. In this study, we applied a two-sample MR method. Through the explanation of the larger proportion of the exposure variance by general instrumental variables (IVs), this method can lead to more accurate causal effect estimation (Bowden et al., 2015; Hartwig et al., 2017). The basic principle of MR analysis for this purpose is shown in Figure 1. According to MR analysis, SNPs influence T2DM outcome through intermediate phenotypes of CRP. Therefore, the SNPs should be significantly associated with CRP but not with T2DM. At the same time, in the population of genes associated with the phenotype, there may be a linkage disequilibrium (LD) effect. Due to the non-random linkage of alleles among different loci in a given population, loci are thought to be in LD when the frequency of association of their different alleles is higher or lower than what would be expected if the loci were independent and associated randomly (Slatkin, 2008; Aissani, 2014). Thus, a notable issue is that the assessment would be biased for those SNPs in LD, since their effects would be expanded when combining all of those SNPs (Noyce et al., 2017).

**FIGURE 2:**
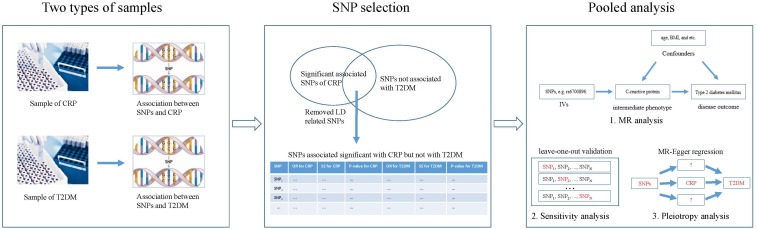
Overview of our method demonstrating the basic ideas for dissecting the influence of CRP on T2DM.

Figure 2 shows the basic idea of dissecting the causal effect of CRP on the risk of T2DM. First, SNP-CRP, and SNP-T2DM association information were extracted from two types of samples, including major and minor alleles for each SNP, minor allele frequency (MAF), standard error (SE), and beta coefficients for each effect allele, both in the T2DM and CRP GWAS databases. Then, suitable SNPs were selected as IVs based on the following principles: (1) SNPs should be significantly associated with CRP but not with T2DM; (2) SNPs with LD should be removed. Finally, a pooled analysis for those IVs based on MR analysis was conducted to evaluate the influence of CRP on T2DM. To assess the effect of a single SNP on the result, a sensitivity analysis was carried out using the leave-one-out method. Pleiotropy in a MR study occurs when the effect of a SNP of IVs on T2DM (the outcome) could be independent of CRP (the intermediate phenotype). Here MR-Egger regression (Bowden et al., 2015) was used to explore the possibility of pleiotropy.

### Samples for Associations Between Genetic Variants and CRP

Significantly associated SNPs of CRP were sourced from (Elliott et al., 2009). They carried out a genome-wide study and a replication study to identify loci associated with plasma CRP concentrations. Their data collection took place from 1989 to 2008, and genotyping occurred from 2003 to 2008. In total, 17,967 participants from five studies (Wyszynski et al., 2005; Bouatia-Naji et al., 2008; Chambers et al., 2008; Yuan et al., 2008; Sabatti et al., 2009) were collected for the genome-wide association study (GWAS), and another 14,747 participants who were not included in the GWAS were collected for the replication study of SNPs associated with CRP. The participants were from West London in the United Kingdom, the Northern Finnish Birth Cohort, the northernmost provinces of Finland, the Lausanne Cohort, and France. All of these participants were 30–75 years of age. Genetic loci associated with CRP were identified from GWAS. The single most closely associated SNP of CRP for each genetic locus was further replicated.

### Samples for Associations Between Genetic Variants and T2DM

Associations between SNPs and T2DM were obtained from the DIAbetes Genetics Replication And Meta-analysis (DIAGRAM) consortium (Zeggini et al., 2008; Lango Allen et al., 2010; Morris et al., 2012), which is a grouping of researchers with shared interest in performing large-scale studies to characterize the genetic basis of T2DM in individuals of European descent. The initial instance of DIAGRAM (DIAGRAM v1) combined data from the Diabetes Genetics Initiative, the Finland-United States Investigation of NIDDM Genetics, and the Wellcome Trust Case Control Consortium (Zeggini et al., 2008; Lango Allen et al., 2010; Morris et al., 2012). An incremental meta-analysis (DIAGRAM v2) added another five GWAS studies of European-descent samples. The recent meta-analysis (DIAGRAM v3) collected 12,171 cases and 56,862 controls.

### SNP Selection

We extracted the significantly associated SNPs (*P*-value < 5 × 10^-8^) of CRP from (Elliott et al., 2009). Then, we extracted the association between these SNPs and T2DM to remove T2DM-associated SNPs (*P*-value < 5 × 10^-2^). The remaining SNPs were further assessed using the web tool SNAP^1^ (Johnson et al., 2008) to remove SNPs in LD (International HapMap Consortium et al., 2007). The remaining SNPs were selected as IVs for further analysis.

### Pooled Analysis

Figure 2 shows the process of pooled analysis based on the selected SNPs (IVs), which includes MR analysis, sensitivity analysis, and pleiotropy analysis.

#### MR Analysis

We first calculated the influence of individual SNPs on T2DM through CRP as individual-level data and then calculated the pooled influence of these individual-level data using the penalized robust inverse-variance weighted (IVW) method (Bowden et al., 2015; Burgess et al., 2016). Assuming *X, Y*, and *Z* are CRP, T2DM, and selected SNPs (IVs), respectively, the Wald ratio (β_*XY*_) of CRP to T2DM for a specified SNP is defined as the following.

(1) $$\beta_{XY}=\beta_{ZY}/\beta_{ZX}$$

where β_*ZY*_ represents the per-allele *log(OR)* of T2DM based on Morris et al.’s (2012) study. β_*ZX*_ is the per-allele *log(OR)* of CRP based on Locke et al.’s (2015) study. In addition, the SE of the BMI-T2DM association of each Wald ratio is defined as the following.

(2) $$SE_{XY}=SE_{ZY}/SE_{ZX}$$

where *SE_ZY_* and *SE_ZX_* represent the *SE* of the variant-T2DM and variant-CRP associations from corresponding studies, respectively. Based on Equations 1 and 2, the individual-level data of the influence of each SNP (IV) on T2DM through CRP was obtained. These data were then analyzed using the penalized robust IVW method (Burgess et al., 2016). The IVW estimate is the same as the two-stage least squares estimate using individual-level data. The penalization of weights from candidate instruments produces heterogeneity in causal estimates, which generates the penalized IVW method, robust IVW method, and penalized robust IVW methods; these methods have been demonstrated to improve the robustness of the findings.

#### Sensitivity Analysis Based on Leave-One-Out Validation

We conducted a leave-one-out validation to test the sensitivity of the selected SNPs (IVs). First, a SNP among the IVs was removed from the IVs to carry out a penalized robust IVW estimate. Then, the fluctuation of the results before and after removing the SNP was observed as the sensitivity. This process was repeated for each of the IVs.

#### Pleiotropy Analysis Using MR-Egger Regression

MR-Egger regression (Bowden et al., 2015), an adaption of Egger regression, was utilized here to detect the directional pleiotropy of IVs. This regression permits variants to show their pleiotropy and assumes that each variant is valid. Only when every variant used for IVs is independent of the alternative path affecting the disease (i.e., horizontal pleiotropy) can yield a consistent estimate via MR-Egger regression (Bowden et al., 2015). The pleiotropy bias of MR analysis is regarded as analogous to small-study bias. Additionally, the average pleiotropic effect across the genetic variants was captured by the estimated value of the intercept in Egger regression. Thus, an intercept that differs from zero is indicative of overall directional pleiotropy.

All the statistical tests for the pooled analysis were undertaken using the R Packages of meta-analysis^2^ and Mendelian Randomization^3^ (Yavorska and Burgess, 2017).

## Results

### IVs for Pooled Analysis

Five SNPs of five genetic loci were significantly associated with CRP (*P*-value < 5 × 10^-8^) based on the previous genome-wide study and the replication study (Elliott et al., 2009). One of them (rs4420638) was associated with T2DM and so was removed. Since the remaining four SNPs were assessed without LD, these SNPs were eventually selected for the pooled analysis in Table 1. Each line of the table documents 13 items involving the SNP, EA and its frequencies, the beta coefficients of the SNP on the risk of BMI and T2DM, and SEs.

**Table 1:** Associations of genetic variants of CRP with T2DM.

SNP	Locus	Chr	BP	EA	NEA	EA freq	Beta CRP	SE CRP	P CRP	Beta T2DM	SE T2DM	P T2DM
rs6700896	LEPR	1	65862370	T	C	0.38	-0.147	0.01429	1.6E–21	-0.00995	0.01493	0.41
rs4537545	IL6R	1	152685503	T	C	0.43	-0.108	0.01531	5.1E–11	-0.01980	0.00991	0.21
rs7553007	CRP	1	157965173	A	G	0.33	-0.207	0.01429	3.3E–38	-0.01980	0.01479	0.14
rs1183910	HNF1A	12	119905190	T	C	0.32	-0.136	0.01429	1.2E–17	0.02956	0.00981	0.059
rs4420638	APOE	19	50114786	G	A	0.19	-0.218	0.01837	2.1E–25	-0.11333	0.02228	2.0E–7


*T2DM, Type 2 diabetes mellitus; EA, effect allele; SE, standard error; EA freq, frequency of effect allele; OR, odds ratio.*

### Pooled Analysis Result

**FIGURE 3:**
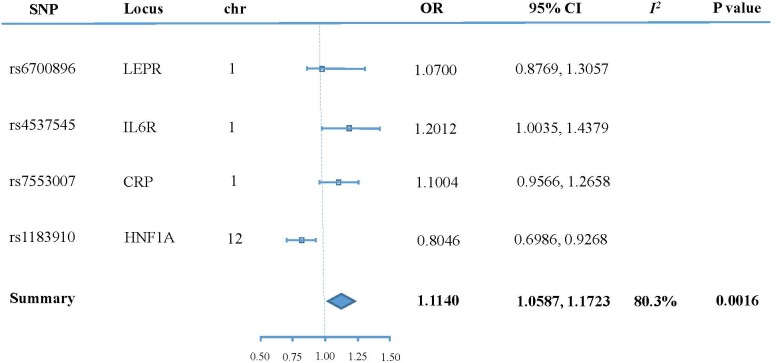
Forest plot of main results.

Figure 3 shows the effect of each SNP and the combined effect of all four SNPs on T2DM through CRP using the penalized robust IVW method. The pooled result (OR 1.114048; 95% CI 1.058656 to 1.172338; *P* = 0.024) showed that high CRP significantly increases the risk of T2DM.

**FIGURE 4:**
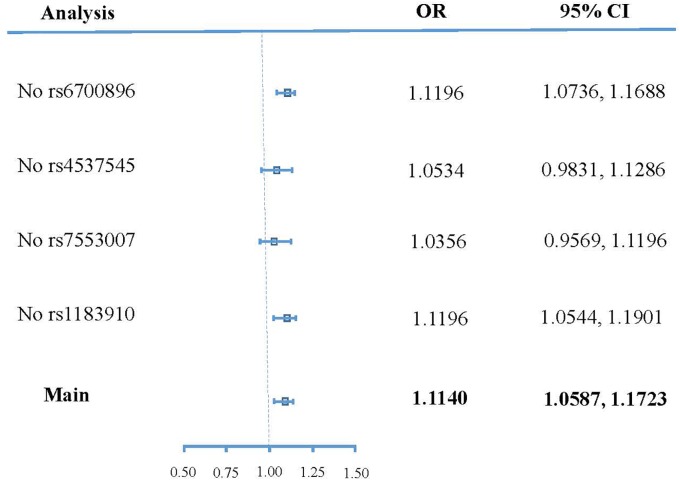
Forest plot of sensitivity analyses.

Figure 4 shows the sensitivity analysis result of SNPs based on leave-one-out validation. The ORs undergo substantial change after removing rs4537545 or rs7553007. In comparison, the ORs undergo a smaller change after removing rs6700896 or rs1183910. These results demonstrated that rs4537545 or rs7553007 drive the penalized robust IVW estimate.

The further estimate of the horizontal pleiotropic effect of these SNPs (IVs) was conducted via MR-Egger analysis. As a result, we got an intercept of 0.017 (95% CI 0.001 to 0.033, *P* = 0.039). This means a potential horizontal pleiotropic effect could bias our estimates.

## Discussion

In this study, we conducted a MR analysis to explore the causal effect of CRP on the risk of T2DM. To reduce the confounding effects, genetic variants were selected as IVs for MR analysis. Five SNPs associated significantly with CRP were extracted from Elliott et al.’s (2009) study. One of them (rs4420638) was associated with T2DM and was removed (Zeggini et al., 2008; Lango Allen et al., 2010; Morris et al., 2012). As a result, the remaining four SNPs without LD were deemed as IVs. The combined effect of the four SNPs on T2DM through CRP (OR 1.114048; 95% CI 1.058656 to 1.172338; *P* = 0.024) was determined using the penalized robust IVW method. This showed that high CRP significantly increases the risk of T2DM.

Brunner et al. (2008) utilized MR analysis for an analogous purpose. They reached the conclusion that associations between CRP and diabetes are likely to be noncausal. In that study, three SNPs were utilized as IVs for MR analysis. The associations between SNPs and CRP were measured on 5,274 men and women, and the associations between SNPs and diabetes were observed among 1,923 patients and 2,932 controls. In comparison, our study included more samples (17,967 and 69,033 participants for measuring SNPs-CRP and SNPs-diabetes associations, respectively). In addition, the diabetes cases in Brunner et al.’s (2008) study include type 1 Diabetes Mellitus (T1DM), T2DM, and others. In contrast, our study focused on T2DM patients. Overall, our study is more specific, and the corresponding conclusion should be more reliable.

To further validate that the different conclusion of Brunner et al. (2008) may be caused by their use of multiple types of diabetes samples, we conducted a MR analysis to evaluate the causal effect of CRP on the risk of T1DM. Associations between SNPs and T1DM were sourced from a meta-analysis of genome-wide association studies with 5,913 T1DM cases and 8,828 reference samples of European ancestry (Barrett et al., 2009), which identified three SNPs (rs6700896, rs7553007, rs1183910) of the five CRP-associated SNPs identified in our study. The associations between these three SNPs and T1DM were then extracted in Table 2 as IVs. The pooled result (OR 1.017145; 95% CI 0.9066489 to 1.14225; *P* = 0.909) did not support a causal role of CRP in the onset of T1DM using the penalized robust IVW method. Therefore, the T1DM samples in Brunner et al.’s (2008) study may be responsible for confusing the causality of CRP in T2DM.

**Table 2:** Associations of genetic variants of CRP with T1DM.

SNP	Chr	BP	EA	NEA	EA freq	Beta CRP	SE CRP	P CRP	Beta T1DM	SE T1DM	P T1DM
rs6700896	1	65862370	T	C	0.38	-0.147	0.01429	1.6E–21	-0.029	0.026	0.2646856
rs7553007	1	157965173	A	G	0.33	-0.207	0.01429	3.3E–38	0.037	0.026	0.1547139
rs1183910	12	119905190	T	C	0.32	-0.136	0.01429	1.2E–17	-0.037	0.027	0.17057


*T1DM, Type 1 diabetes mellitus; EA, effect allele; SE, standard error; EA freq, frequency of effect allele; OR, odds ratio.*

The advantage of MR analysis is that no confounding factor should be considered when using genetic variants as IVs, since the genetic variants are free and unaffected by confounding factors. In comparison, confounding factors can heavily affect the results of observational studies. Therefore, most of these observational studies should be adjusted for potential confounding factors. However, abundant phenotypes of T2DM make it hard to do so. This issue occurs in Lee et al.’s (2009) prospective study about whether adiponectin is a confounder. In their study, observed that CRP was associated with T2DM after adjusting for age, sex, body mass index (BMI), etc. However, the association was completely attenuated after further adjustment for serum adiponectin. The recent evidence suggests that CRP inhibits adiponectin gene expression in adipocytes (Yuan et al., 2007). If so, adjustment for adiponectin would be over-adjustment.

Current studies on the treatment of T2DM have also reflected the potential causal effect of CRP on the risk of T2DM. During the treatment of T2DM patients with Metformin and Silymarin, researchers observed a significant decrease of CRP levels (Koujan et al., 2015; Shi et al., 2016). They even used these decreased serum levels of CRP as a sensitive predictor in T2DM patients being treated with drugs (Koujan et al., 2015; Shi et al., 2016). To explore effective curative options for T2DM, some researchers mainly refer to the effect of treatment on the serum CRP levels of T2DM patients. Kim (2014) observed that training modes can help to decrease the serum CRP level of T2DM patients. Rutten et al. conducted intensive care and routine care on 235 and 189 T2DM patients, respectively. A significant decrease of CRP levels based on intensified multifactorial treatment was observed in T2DM patients after six years (den Ouden et al., 2015).

The incidence of T2DM can be associated with multiple phenotypic factors. The potential linkages among these factors can help us to comprehensively understand the mechanisms of T2DM. The role of CRP on the risk of T2DM necessitates a measurable effect for us to mine these linkages. To the best of our knowledge, CRP is an annular, pentameric protein in blood plasma, the levels of which rise in response to inflammation. Thus, long-term inflammation can also increase the risk of T2DM through CRP. In the previous MR analysis, body mass index (BMI), and waist circumference (WC) are two immediate phenotypes of T2DM (Corbin et al., 2016). Stronger associations between these two phenotypes and CRP were observed in a recent study (Priyanka et al., 2013). This means that either CRP affects T2DM through BMI and WC, or BMI and WC affect T2DM through CRP.

Although our samples were restricted to those of European ancestry and IVs with LD were removed to reduce bias, our analysis also has limitations worth consideration. First, IV-CRP and IV-T2DM associations were sourced from two different samples. In theory, a single sample source may be more reliable than multiple sample sources. Fortunately, the increase of the number of samples reduces this type of bias. Next, horizontal pleiotropy of IVs may influence the conclusion of MR method. Here we evaluated the relevance between genes that IVs were located in (Table 1) with T2DM using an enrichment tool BLAT2DOLite (Cheng et al., 2016). No associations were detected, which means that these IVs cannot impact T2DM directly. Nevertheless, they could take effect in other immediate phenotypes of T2DM, such as BMI, WC, etc. The lack of associations between IVs and other phenotypes limits our further validation. Finally, without a specified indication, these results are ill-suited for clinical application.

In summary, we validated that the increase of CRP levels enhances the risk of T2DM and has no effect on T1DM using MR analysis. Here, genetic variations are deemed as IVs to reduce the potential confounding effect. The further validation relies on RCTs. The result has a very positive guiding role in finding new therapeutic strategies and therapeutic targets. This is related to the early prevention of T2DM, which has the potential to indirectly, from the intervention phenotype, regulate the development of disease. In the future, we will construct a dataset including the relationships between genetic variations and the phenotypes of DM. The data set may be beneficial in designing computational tools for phenotype interaction (Peng et al., 2017, 2018a) and function association prediction (Cheng et al., 2018b; Hu et al., 2018; Peng et al., 2018b).

## Author Contributions

LC, HJ, JZ, and SW conceived and designed the experiments. LC, HZ, and SY analyzed the data. LC wrote the manuscript. All authors read and approved the final manuscript.

## Conflict of Interest Statement

The authors declare that the research was conducted in the absence of any commercial or financial relationships that could be construed as a potential conflict of interest.

## Footnotes

1 https://ldlink.nci.nih.gov/?tab=snpclip

2 http://cran.r-project.org/web/packages/meta/index.html

3 https://cran.r-project.org/web/packages/MendelianRandomization/
