# Corrigendum: Exposing the Causal Effect of C-Reactive Protein on the Risk of Type 2 Diabetes Mellitus: A Mendelian Randomization Study

**DOI:** 10.3389/fgene.2019.00085

**Published:** 2019-02-18

**Authors:** Liang Cheng, He Zhuang, Shuo Yang, Huijie Jiang, Song Wang, Jun Zhang

**Affiliations:** ^1^College of Bioinformatics Science and Technology, Harbin Medical University, Harbin, China; ^2^Department of Radiology, The Second Affiliated Hospital of Harbin Medical University, Harbin, China; ^3^Department of Radiology, Longhua Hospital, Shanghai University of Traditional Chinese Medicine, Shanghai, China; ^4^Department of Rehabilitation and Pharmacy, Heilongjiang Province Land Reclamation Headquarters General Hospital, Harbin, China

**Keywords:** C-reactive protein, type 2 diabetes mellitus, causal effect, genome-wide association studies, mendelian randomization, type 1 diabetes mellitus

In the original article, we neglected to include the funder “Scientific Research Project of Health and Family Planning Commission of Heilong Jiang Province, 2018415” to “Huijie Jiang.”

Additionally, in the published article, there was an error in affiliations 2 and 3. Instead of affiliation 2, “Department of Radiology, Shanghai University, Shanghai, China,” it should be “Department of Radiology, The Second Affiliated Hospital of Harbin Medical University, Harbin, China,” and instead of affiliation 3, “Heilongjiang Provincial Hospital, Harbin, China,” it should be “Department of Radiology, Longhua Hospital, Shanghai University of Traditional Chinese Medicine, Shanghai, China.”

In the published article, there was an error regarding the affiliation for Jun Zhang. He should have the affiliation 4 “Department of Rehabilitation and Pharmacy, Heilongjiang Province Land Reclamation Headquarters General Hospital, Harbin, China.”

The authors apologize for these errors and state that this does not change the scientific conclusions of the article in any way. The original article has been updated.

